# First-in-line 3D-printed intravaginal ring for nonhormonal contraception

**DOI:** 10.21203/rs.3.rs-7935107/v1

**Published:** 2025-11-21

**Authors:** Sarah Anne Howard, Maimoona S Bhutta, Rima Janusziewicz, Andrés Prieto Trujillo, Ava L. Cohen, Gustavo F. Doncel, S. Rahima Benhabbour

**Affiliations:** 1 Division of Pharmacoengineering and Molecular Pharmaceutics, UNC Eshelman School of Pharmacy, University of North Carolina at Chapel Hill, Chapel Hill, NC, USA; 2 CONRAD, Eastern Virginia Medical School, Macon & Joan Brock Virginia Health Sciences at Old Dominion University, Norfolk, VA, USA; 3 Lampe Joint Department of Biomedical Engineering, North Carolina State University, Raleigh, NC and the University of North Carolina, Chapel Hill, NC

**Keywords:** Nonhormonal contraception, 3D printed IVR, long-acting delivery, sperm motility, spermicidal activity

## Abstract

With nearly half of all pregnancies occurring unintended, effective and acceptable contraceptive options remain a global necessity. Though contraceptives with extended durations of action reduce the need for strict daily adherence, thus enhancing compliance and reliability, only one of these methods – the copper intrauterine device (IUD) – prevents pregnancy without administering exogenous hormones. Herein, we demonstrate co-delivery of two nonhormonal contraceptive agents, lactic acid (LA) and glycerol monolaurate (GML), using a next-generation 3D-printed intravaginal ring (IVR). Through alterations in ring properties and drug loading, a range of LA and GML release rates were achieved *in vitro*, demonstrating the flexibility of the platform technology. Rings elicited sustained release of LA and GML at target release rates over 30 days or longer. Additionally, these studies explored how drugs with different physiochemical properties interact within the IVR matrix and further elucidated IVR drug loading and release mechanisms.

## Graphical Abstract

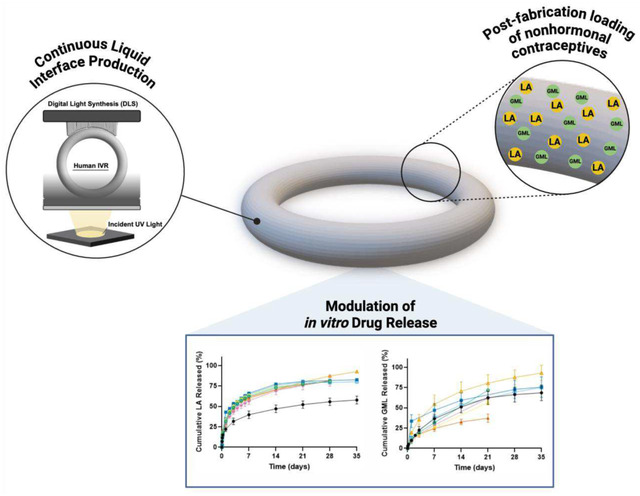

## 1. INTRODUCTION

While the landscape of female-based contraceptive options may seem expansive, the most ones are variations of hormonal methods. In the United States, the contraceptive injectable^1^, implant^2^, “minipill^3,4^,” and hormonal intrauterine device (IUD)^5^ options all provide birth control by dosing synthetic progestogens. Combinations of synthetic progestogens and estrogens can be delivered for contraception in a daily oral pill^3,6^ or a 21-day intravaginal ring (IVR).^7,8^ In contrast to the options listed above, only the Copper IUD enables continuous, yet reversible, contraceptive protection without the systemic dosing of exogenous hormones.^9,10^ This reveals a gap in nonhormonal contraceptives for a user-controlled option that provides continuous protection.^11^ User-controlled options do not rely on a healthcare provider or other professional to initiate or terminate a contraceptive regimen. These methods enhance accessibility and enable autonomy in reproductive decisions. Intravaginal rings are acceptable and established as contraceptive devices due to their inconspicuous nature, user-driven application, and extended durations of continuous protection between 1 and 12 months.^12–18^

Traditional IVR manufacturing utilizes hot-melt extrusion to mix a polymer with the active pharmaceutical ingredients (APIs), followed by injection molding to produce solid rings.^19^ This process has several disadvantages, including narrow API compatibility due to the inherent high temperatures and pressures, limited size and design flexibility with expensive retooling costs, and substantial waste as incomplete drug release necessitates overloading the IVRs with APIs to achieve target release rates and durations.^19,20^ As an alternative, this study leverages a unique 3D-printing process called **c**ontinuous **l**iquid **i**nterface **p**roduction (CLIP^™^).^21–23^ 3D-printing enables geometric complexity beyond the solid rings produced by traditional manufacturing. This geometric complexity can be used to fine-tune drug release by altering ring surface area and diffusion distances.^24,25^ Furthermore, computer-aided design (CAD) allows for new ring types with varied geometric complexity to be produced rapidly at low cost. As typical 3D-printing is performed in a layer-by-layer process, this could result in IVRs with a rough, ridged, and irritating exterior.^26,27^ Therefore, this study leverages the mechanisms of CLIP 3D-printing to produce monolithic rings with a smooth surface.^21,22^ CLIP uses UV photopolymerization of a liquid resin to fabricate customizable devices in a fast and scalable process.^21,23–25^ Uniquely, this process temporarily inhibits photopolymerization by way of an oxygen-rich “dead zone” which enables a continuous, rather than layer-by-layer, production.^23^

This proof-of-concept work seeks to highlight the potential for a novel nonhormonal and user-controlled reversible contraceptive using 3D-printed IVRs. While systemic circulation is necessary for protection with hormonal contraceptives,^28–30^ we sought to investigate locally-acting contraceptive agents for the development of a nonhormonal concentrative IVR. As a result, we identified and selected lactic acid (LA) and glycerol monolaurate (GML, or monolaurin) as two potential nonhormonal contraceptive compounds.

Lactic acid, an active ingredient of the FDA-approved nonhormonal contraceptive gel Phexxi^®^ and an endogenous compound present in the female reproductive tract, can be utilized to acidify the vaginal environment.^31,32^ The typical human vaginal pH of 4–5 is too acidic for the survival and function of sperm.^33–35^ To this end, semen acts to temporarily buffer the vagina to a more neutral pH, thus enabling sperm viability and motility and ascend through the reproductive tract. As such, the use of LA to maintain the acidic vaginal environment is aimed to sustain a vaginal pH inhospitable to sperm.

GML is a fatty acid found in coconut oil with natural anti-inflammatory and antibacterial properties.^36,37^ It has also been shown to be virucidal, capable of inhibiting HIV-1 and other viruses.^38–41^ Previous work by Ball *et al*. demonstrated that GML was also capable of producing contraceptive effect by inhibiting sperm motility and viability.^42^ As such, the combination of GML with LA in our nonhormonal IVR could provide a synergistic effect to support efficacy with two mechanisms of contraceptive action.

With this nonhormonal combination drug regimen, we assessed the ability to homogenously load a hydrophilic (LA) and hydrophobic (GML) compounds using our single step proprietary drug loading process^25^ and their *in vitro* release, stability, effects on sperm immobilization and reversibility, effect on sperm motility, and *in vitro* cytotoxicity using cell-based assays to determine the safety of LA and GML on human vaginal epithelial cells. Collectively, we demonstrated the ability to develop a 3D-printed NH contraceptive IVR in a unique and biocompatible silicone-based material, incorporated two APIs using a novel single-step post-fabrication loading method, and showed sustained release of LA and GML *in vitro* with evidence of a potential synergistic effect on sperm motility. We also showed that GML elicited a dose-dependent cytotoxicity on human vaginal epithelial cells. Future studies will assess the safety of both LA and GML in cell lines or tissue explants representative of the female genital tract (FGT) and *in vivo* safety, pharmacokinetic, and efficacy studies in the sheep model.

## 2. MATERIALS AND METHODS

### 2.1 Materials

Intravaginal rings were fabricated with SIL 30 resin, provided in kind by Carbon, Inc., using an M1 digital light synthesis printer (Carbon, Inc.; Redwood City, CA). Glycerol Monolaurate (GML, Monolaurin) was purchased from Tokyo Chemical Industry Co. (TCI; Tokyo, Japan, Cat. No. G0081) and lactic acid (LA, DL-Lactic acid) was purchased from Fisher Chemical (Cat. No. A159). Ethanol (EtOH, 200 Proof) was purchased from Decon Laboratories Inc. (Cat. No. 2705HC). Methanol (MeOH, Cat. No. A412–4) was obtained from Fisher Chemical. Fluorescent dyes Nile Blue A (NBA, Cat. No. N0766) and Rhodamine B (RhB, Cat. No. R6626) were purchased from Sigma. Potassium phosphate monobasic (Fisher Chemical, Cat. No. P285) was used to prepare a 10 mM solution of phosphate buffer and adjusted to pH 2.4 using phosphoric acid. A 25 mM solution of acetic acid was prepared with sodium acetate (Fisher Chemical Cat. No. S210) and adjusted to pH 4.2 using 1N NaOH and utilized as simulated vaginal fluid (SVF) for *in vitro* release studies.

### 2.2 Quantitative Analyses of Lactic Acid and Glycerol Monolaurate

A quantitative reverse-phase HPLC method was developed for lactic acid analysis using an Agilent 1260 Infinity II system, equipped with a photodiode-array detector, and a Restek Ultra Aqueous C18 column (5 μm, 150×4.6 mm, Cat #9178565). After method development, samples were diluted 1:1 with 10 mM phosphate buffer (pH 2.4) for quantification. Phosphate buffer was prepared by dissolving 5.44 g of potassium phosphate monobasic in 4L of HPLC-grade water and adjusting to pH 2.4 with phosphoric acid. Volumes of 10–25 μL were injected by an autosampler and chromatographically separated using an isocratic mobile phase of 10 mM phosphate buffer at a rate of 0.8 mL/min. Absorbance signals were read at a wavelength of 210 nm. The lower limits of detection and quantification were 2.55 and 8.51 μg/mL, respectively.

Glycerol monolaurate was quantified by liquid chromatography-mass spectrometry (LC-MS). Analyses were performed using a Finnigan LTQ Ion Trap (Thermo Fisher Scientific Inc.) equipped with a Acquity UPLC sytem (Waters^™^). A validated gradient method was created using mobile phases including water with 0.1% formic acid and acetonitrile with 0.1% formic acid. Samples were diluted in MeOH and volumes of 10–25 μL were injected by an autosampler for chromatographic separation with a Symmetry C18 column (Waters, 3.5 μm, 100×2.1 mm) stationary phase using a flow rate of 0.4 mL/min. Samples were analyzed in positive ion mode across a scan range of 140–2000 m/z.

### 2.3 Determination of Saturation Solubility

Saturation solubility of GML was determined in relevant solvents (Water, Acetone, MeOH, EtOH) and *in vitro* release media (SVF with 2% Solutol). Approximately 20 mg samples of GML were weighed into microcentrifuge tubes and 100 μL of solvent (n=3) was subsequently added. Samples were vortexed and assessed visually to determine dissolution progress. As dissolution occurred, an additional 20 mg of GML was added until complete saturation was reached. All samples were then stored, protected from light, overnight to confirm complete saturation. After 24 hours, samples were centrifuged for 12 min at 16,000g (Eppendorf Centrifuge 54145R, USA) and aliquots (n=3) of saturated supernatant were collected for analysis. The supernatant samples were diluted with MeOH and drug concentration was subsequently analyzed via LC-MS as described above.

### 2.4 Determination of LA Target Dose by Titration and Cell-based Assays

#### 2.4.1 Lactic Acid Titration

The amount of lactic acid necessary for contraceptive efficacy was estimated by measuring solution pH. Simulated vaginal and seminal fluids, with physiologically-representative buffer capacities, were prepared as previously described by Rastogi *et al*.^43^ Simulated vaginal fluid (SVF) was mixed with seminal fluid simulant (SFS) in a 1:5 ratio to represent potential fluid volumes during intercourse. Lactic acid was added gradually to both the individual simulated reproductive fluids and the 1:5 fluid mixture. The pH of each solution was monitored following each addition of lactic acid using a 3-point calibrated pHTestr^®^50S probe (Oakton Instruments).

#### 2.4.2 Modified Sander-Cramer Assay for Spermicidal Activity

Semen samples were collected from consented healthy male donors by masturbation after 2–5 days of sexual abstinence. The protocol was approved by the Eastern Virginia Medical School’s Institutional Review Board (IRB) at the Macon & Joan Brock Virginia Health Sciences, Old Dominion University. Semen analysis was performed according to WHO guidelines.^44^

The highest spermicidal dilution and minimum effective concentration (MEC) for the test compounds were determined using the modified Sander-Cramer (SCr) assay.^45^ Serial dilutions of lactic acid and glycerol monolaurate were prepared in 0.9% saline, immediately before the addition of semen. Briefly, 250 μL of test compounds were incubated with 50 μL of semen for 30 sec at room temperature (RT). During the incubation period, each sample was examined under a phase-contrast microscope (E600 Nikon Eclipse, Melville, MY) for the presence of motile sperm. To evaluate the protective effect of seminal plasma, 50 μL of test compounds were incubated with 50 μL of semen (1:1 dilution ratio) for 30 sec at room temperature (RT). If any motile sperm (i.e. progressive, twitching, etc.) were found within 30 seconds, then the dilution was recorded as ‘Failed’. If no motile sperm were found, the dilution was considered as ‘Pass.’ The results from 4–6 independent experiments were combined, and the highest spermicidal dilution and MEC were calculated as $MEC=\frac{\text{initial}\;\text{concentration}}{\text{highest}\;\text{spermicidal}\;\text{dilution}}$ and presented as Mean ± SE.

To see whether the sperm immobilizing effect of the compounds was reversible, 1 mL of warmed Ham’s F-10 media (Millipore Sigma, MA, USA) supplemented with 1% Human Serum Albumin (HSA) (Millipore Sigma, MA, USA) was added to each sample to further dilute the compound concentration and assess the reversibility of the sperm immobilization effect. Sperm were incubated for an additional 60 mins at 37°C. After this sperm “recovery” period, motility was re-evaluated with manual counting and calculated as:

$$\%\text{Sperm}\;\text{Motility}=\frac{\text{Total}\;\text{Motile}\;\text{Sperm}}{\text{Total}\;\text{Motile}+\text{immobile}\;\text{Sperm}}*100.$$

#### 2.4.3 Time-Response Sperm Motility Assay

A time-response experiment was conducted to assess the impact of contraceptive compounds on sperm motility at various time points following incubation. Sperm samples were incubated with varying concentrations of LA or GML (1:1 dilution ratio) for 30 secs, 10, 30, 60, and 120 minutes. Concentrations of GML were selected based on the critical micelle concentration (CMC) of 16.4 μg/mL.^43^ Each sample was examined under a phase-contrast microscope, and sperm motility was assessed manually.

Combining compounds with different mechanisms of action can increase the overall treatment efficacy. The combined impact of GML and LA on sperm motility was examined with a time-response assay. Sperm samples were incubated with suboptimal concentrations of LA (5 mg/mL) and GML (0.02 or 0.2 mg/mL) for 30 sec, 2, 10, 30, and 60 minutes, respectively. Each sample was examined under a phase-contrast microscope, and sperm motility was assessed via manual counting.

#### 2.4.4 In vitro Cytotoxicity Assessment

Human vaginal epithelial cells, VK2/E6E7 (ATCC^®^ CRL-2616^™^), were used to evaluate GML’s effect on cell viability. VK2 cells were propagated in Gibco^™^ Keratinocyte SFM (1×) media (Cat# 17005042, ThermoFisher Scientific, MA, USA) supplemented with 0.1 ng/mL epidermal growth factor, 50 μg/mL bovine pituitary extract, 0.4 mM CaCl2, and 1% penicillin and streptomycin (P/S; Cat# 15140–122, Gibco). Cells were incubated with varying concentrations of GML for 24 hrs at 37°C. Cell viability was assessed using CellTiter 96^®^ AQueous Non-Radioactive Cell Proliferation (MTS) Assay (Cat# G5421, Promega, WI, USA) per manufacturer instructions.

### 2.5 Fabrication of 3D-Printed IVRs with Continuous Liquid Interface Production (CLIP^™^)

Intravaginal rings were 3D-printed by Continuous Liquid Interface Production (CLIP^™^) using SIL 30, a dual-cure silicone polyurethane resin (Carbon Inc.). Three different ring types were fabricated for this study. A variety of ring types were designed with specific dimensions, print parameters, and geometric complexity using CAD and exported as an .STL file for use. Files were prepared to vertically fabricate 14–16 rings at a time using an M1 printer (Carbon Inc.). For each ring batch, SIL 30 resin (190–208 g) was dispensed to fill the reservoir cassette. The M1 printer selectively projects light through an oxygen permeable window on the reservoir cassette according to the provided .STL file for the initial cure and resin solidification through photopolymerization. At the conclusion of each print, IVRs were removed from the build platform, separated from their supports, and smoothed using a blade or a custom-made 3D printed smoothing tool. Smoothed IVRs were then placed in isopropyl alcohol (IPA) for a 2-minute dynamic wash. IVRs were allowed to briefly air-dry before undergoing an 8-hour thermal process to initiate a secondary amine reaction.^46^ The physical metrics of 3D-printed IVRs, including mass, outer-diameter, and cross-sectional diameter were measured. IVRs were stored at 4°C prior to drug loading.

### 2.6 Drug Loading by Post-Fabrication Swelling

GML and LA were homogenously incorporated within the 3D printed IVRs via a solvent-mediated process. The silicone polyurethane material utilized in the IVRs, SIL 30 (Carbon Inc.), possesses a marked ability to swell in organic solvents. Therefore, IVRs were placed in ethanol-based solutions containing both GML and LA (hereafter referred to as loading solutions) for 48 hours to allow equilibrium swelling and maximize drug absorption and distribution homogeneity within the IVR matrix. Drug-loaded rings were subsequently air dried to allow for evaporation and complete removal of residual ethanol.

### 2.7 Development of Weight-Based Linear Loading Equations

The amount of drug absorbed and incorporated within the intravaginal rings during the post-fabrication swelling process is directly related to the concentration of drug in the loading solution.^21,25,47–49^ Linear equations were developed for each ring type utilized to correlate the concentrations of GML and LA in the loading solutions to the weight percent of drug loading as defined by the mass of loaded drug divided by the initial weight of the fabricated IVR. For each ring design, a concentrated solution containing both GML and LA in ethanol was prepared and serially diluted to generate five dual-drug loaded solutions (300 mL). Previously weighed IVRs were placed in each successively diluted solution (n=3 each solution, each ring type) and allowed to swell for 48 hours. After the swelling period, IVRs were air-dried before determining the post-loading mass of each ring. The loaded amounts of GML and LA were determined by extracting the drugs from the rings. Extractions were performed by placing drug loaded rings in individual jars containing 100 mL of ethanol for 24–48 hours. Aliquots (1 mL, n=3) of each solution were sampled before replacing the ethanol and repeating the extraction until completion. Samples were analyzed to quantify the total amount of GML and LA per ring as described previously. The weight percent of each drug was determined for each solution concentration and loading equations were then generated via linear regression. The linear loading equations were utilized to determine the appropriate loading solution concentrations necessary to achieve target drug loadings.

### 2.8 In vitro Drug Release Studies

*In vitro* release studies were completed to determine drug release kinetics of GML and LA for each ring design. Drug loaded IVRs (n=3 each) were placed in individual jars and incubated in simulated vaginal fluid (200 mL SVF, 25 mM sodium acetate with 2% solutol, adjusted to pH 4.2) at 37°C under dynamic agitation at 120 rpm using a benchtop incubator shaker. At predetermined timepoints, aliquots (1 mL, n=3) of release media were collected and replaced with an equivalent volume of fresh SVF. Complete replacement of release media was performed regularly to ensure studies remained under sink conditions, maintaining drug concentrations at or below 1/5^th^ their maximum solubility in SVF (i.e. ≤ 650 μg GML / mL). The samples were analyzed using HPLC and LC-MS, using the aforementioned methods, to quantify cumulative drug release.

### 2.9 Effects of Partial Drug Unloading on Initial Burst Release

The concept of partial drug unloading from IVRs was explored to reduce the initial burst release, particularly for LA. Thirty-six (36) rectangular blocks of SIL 30 resin (10 × 20 × 7.6 mm^3^) were fabricated as described in Section 1.2.5 to model the swelling abilities of IVRs.^25^ A concentrated ethanol solution containing GML (114.1 mg/mL) and LA (33.8 mg/mL) was prepared and serially diluted to make three loading solution concentrations. Solutions were analyzed to quantify GML and LA analytical concentrations as previously described in Section 1.2.2. Blocks (n=12 each) were placed in each loading solution and allowed to swell for 48 hours to reach equilibrium swelling. After swelling, blocks were removed from the loading solutions and allowed to dry completely. Three (3) blocks from each loading solution were placed in neat ethanol for 1, 3, or 24 hours to partially unload the drugs and mimic the burst release. Following a second drying period, blocks were placed in SVF (15 mL) and incubated at 37°C to monitor drug release over one week as described in Section 1.2.8. Upon culmination of the week-long release monitoring, the remaining drug in each block was extracted in successive 15 mL volumes of EtOH until completion.

To allow a visual representation of the drug loading and unloading process, solid IVRs were loaded with representative dyes. A hydrophobic dye, rhodamine B (RhB) and a hydrophilic dye, Nile Blue A (NBA), were utilized to model lactic acid and glycerol monolaurate, respectively. Dyes were loaded individually and in combination via post-fabrication swelling in ethanol solutions. Aliquots (1mL, n=3) of each initial dye solution were sampled and stored at 4°C prior to analysis. After swelling, rings were photographed before incubating them in neat ethanol for 1, 3, or 24 hours to partially unload the dyes. After the unloading period, rings were photographed and sample aliquots (1 mL, n=3) of unloaded solvent were collected. The concentrations of RhB and NBA in each sample were determined by quantifying fluorescent intensity using a Synergy H1 microplate reader (BioTek) at 530/560 nm and 630/660 nm Ex/Em, respectively. Duplicate standard curves were prepared in ethanol and samples (200 μL) were analyzed in triplicate, diluting in ethanol if necessary. Dye concentrations were quantified by interpolation of each standard curve using Graphpad PRISM.

## 3. RESULTS AND DISCUSSION

### 3.1 Quantitative Analyses of Lactic Acid and Glycerol Monolaurate

The physiochemical properties of non-hormonal contraceptive compounds, LA and GML, influence their interaction with the SIL-30 polymer matrix and their release into the aqueous environment.^21,25,48^ As such, robust analytical techniques to quantify GML and LA were developed to evaluate these potential interactions on LA and GML release kinetics *in vitro*. Due to the absence of a chromophore in GML, ultraviolet (UV) detection by a diode array detector (DAD) using high performance liquid chromatography (HPLC) was not possible. Therefore, liquid chromatography-mass spectrometry (LC-MS) was utilized to detect and quantify GML. A traditional gradient method was developed (Supplemental Table 1) to detect GML in positive ion mode at 276 m/z as it eluted after approximately five minutes using a 0.4 mL/min flow rate.

In contrast, the UV-absorption of LA was easily detected at 210 nm using HPLC equipped with a DAD. Given the acetic acid in the SVF utilized as release media, a highly specified method needed to be established for the clear separation of the two similar compounds **(Fig. 1)**. An aqueous mobile phase consisting of 10 mM phosphate buffer (pH 2.4) was utilized to maintain ionization of LA based on previous analytical HPLC methods described in the literature.^50–52^ Initially, an isocratic method *(Method 1)* was developed using 90% 10 mM phosphate buffer (A) and 10% acetonitrile (B) at a flow rate of 1 mL/min. Given the high aqueous content of the mobile phase, an Ultra AQ C18 column (Restek) was selected for improved performance and stability over traditional C18 columns. This initial method, however, was not capable of properly separating lactic acid from the acetic acid in SVF. A second method (*Method 2*) was developed using 100% 10 mM phosphate buffer, which resulted in complete separation of LA and acetic acid; however, the LA peak displayed an undesired shoulder next to the main peak. It was hypothesized that the shoulder could be a result of solvent mixing between the SVF (pH 4.2) and the 10 mM phosphate buffer (pH 2.4). Therefore, the amount of SVF injected on each run was reduced with two potential solutions: 1) diluting SVF samples 1:1 with 10 mM phosphate buffer (*Method 3*) or 2) lowering injection volume from 25 μl to 10 μl (*Method 4*). Additionally, to ensure good resolution between the peaks these methods were operated at a reduced flow rate of 0.8 mL/min from 1 mL/min. Both solutions provided complete separation of lactic acid and acetic acid with good-quality peaks and resolution >2.4. The resultant standard curve is shown in Fig. 1C.

### 3.2 Determination of Saturation Solubility

Once analytical methods were developed, the saturation solubility of GML and LA in relevant solvents was determined using the optimized methods described above. The silicone polyurethane matrix of our CLIP 3D-printed IVRs swells significantly in organic solvents such as EtOH and acetone. This unique matrix swelling ability in biocompatible organic solvents is leveraged for drug loading using a controlled absorption process.^25,48,49^ The saturation solubility of LA and GML in EtOH and acetone was determined to identify the preferred solvent to maximize solution concentration and subsequent drug loading capacity in IVRs. Furthermore, saturation solubility in water and release media (SVF) was determined to ensure sink conditions were maintained throughout the *in vitro* release study duration. LA, utilized in its liquid state, was miscible with all tested solvents. GML elicited good solubility in the organic solvents, but low aqueous solubility (Table 1). Ethanol, a Class 3 residual solvent,^53^ was selected for post-fabrication drug loading based on the higher drug solubility and the higher IVR equilibrium swelling (Table 1).

### 3.3 Target Development by Titration and Cell-based Assays

As sperm viability decreases in acidic environments, seminal fluids are necessary to neutralize the vaginal pH and enable fertilization.^35,54^ While both vaginal fluid and seminal fluid are capable of pH buffering, seminal fluid’s greater buffering capacity ensures that pH neutralization briefly occurs.^43^ Localized acidifying agents, such as LA, can be utilized to help maintain an acidic vaginal pH even in the presence of seminal fluids.^31^

Target intravaginal delivery doses of LA were estimated by measuring pH following gradual additions of LA to a 1:5 mixture of SVF and SFS with buffer capacities comparable to human samples (Fig. 2).^43^ Changes in pH were also measured after adding LA to each simulated fluid independently. Initially, the SVF had an average pH of 4.14 and the SSF had an average pH of 7.65. After mixing the fluids in a 1:5 ratio to replicate potential vaginal conditions after intercourse and ejaculation,^55–58^ the average measured pH of the resulting mixture was 6.94. The mixture pH reached 4.2, typical human vaginal pH, once LA concentration reached 24 mM (2.16 mg/mL). At the same LA concentrations, SVF alone reached a pH of 3.67 while SSF alone reached a pH of 3.37. As a result, an initial target daily dose of LA between 14–45 mg/day was established to offer efficacious buffering without over-acidification.

The initial target daily dose of GML was established at 2.5–25 mg/day by scaling the efficacious concentrations of GML observed to cause sperm (6 mL) to lose motility for clinical efficacy as previously reported by Ball *et al*.^42^ IVRs were fabricated and loaded with LA and GML to achieve these initial targets (14–45 mg/day LA and 2.5–25 mg/day GML). Using cell-based assays, LA and GML release rates were optimized based on safety and efficacy outcomes. Using established LA and GML loading equations, IVRs were subsequently loaded with LA and GML doses to achieve the optimized *in vitro* release rates over 28 days.

#### 3.3.1 Sperm Immobilizing Activity of LA and GML

**Figure 3.**
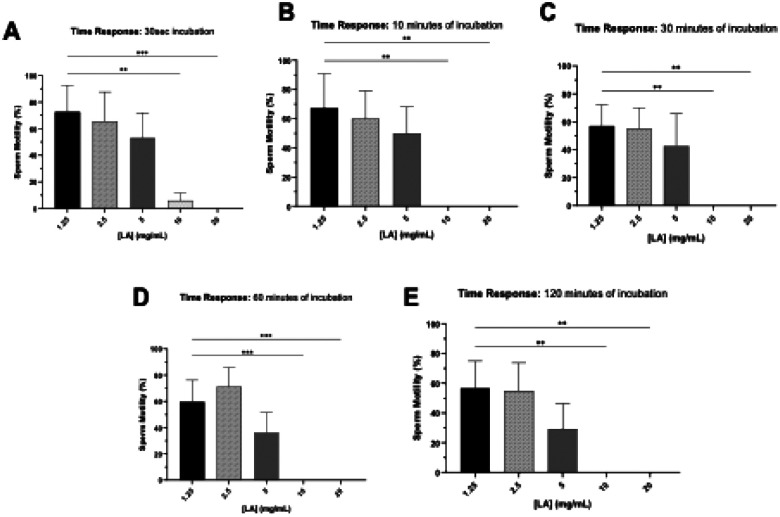
Time response assessment of sperm immobilizing effect of LA. Time-response experiment assessing the sperm immobilization activity of LA at 30 sec, 10, 30, 60, and 120 mins. Sperm motility was assessed by manual microscopic counting. Statistical analyses were conducted using one-way ANOVA with Tukey’s multiple comparison tests. All error bars represent SD (n=3). *p< 0.05, ** *p*< 0.01, *** *p*< 0.001, **** *p*< 0.0001.

**Figure 4.**
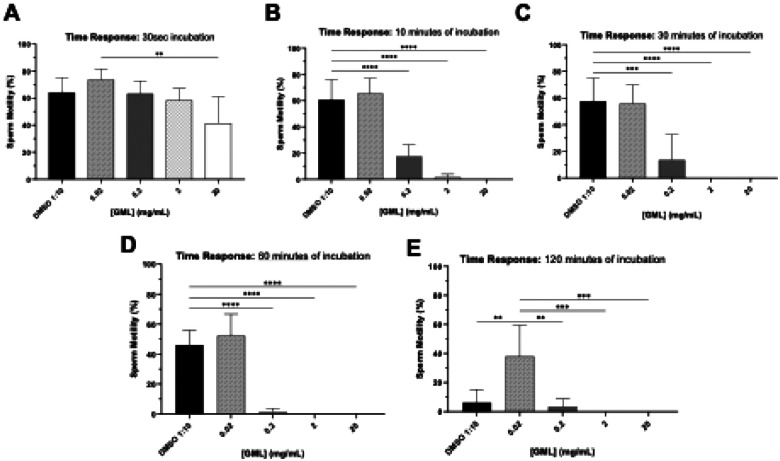
Time response assessment of sperm immobilizing effect of GML. Time-response experiment assessing the sperm immobilization activity of 0.02, 0.2, and 20 mg/mL of GML at 30 secs, 10, 30, 60, and 120 mins. Sperm motility was assessed manually by microscopic counting. Statistical analyses were conducted using one-way ANOVA with Tukey’s multiple comparison tests. All error bars represent SD (n=4). * *p*< 0.05, ** *p*< 0.01, *** *p*< 0.001, **** *p*< 0.0001. DMSO 1:10 (v/v) is the diluent control for 20 mg/mL GML.

Utilizing the gold-standard Sander-Cramer (SCr) assay,^59^ the minimum effective concentration (MEC) of LA following 30 sec exposure was calculated as 9 mg/mL. The MEC of LA did not change during the recovery period (Table 2, Fig. 3). GML had a dose-dependent effect on sperm motility following 30 sec incubation (p<0.001, Fig. 4). The MEC of GML was calculated as 45 ± 5.5 mg/mL; however, the recovery MEC of GML following 60 mins drastically decreased to 1.7 ± 0.9 mg/mL, as most of the sperm cells remained immobilized (Table 2, Fig. 4). At concentrations above 0.2 mg/mL, GML visibly precipitated in 0.9% saline. N-9, a well-known spermicide, was used as the experimental positive control (Supplementary Figure 1).

When concentrations of LA or GML were incubated with semen in a 1:1 dilution ratio, amplifying the potential protective effect of seminal plasma, the MEC (in modified SCr assay) of LA and GML following 30-sec exposure and follow-on dilution and 60 min incubation increased to 18 mg/mL (13 ± 3.5 mg/mL post-dilution) and 55 ± 5.8 mg/mL (1.7 ± 0.9 mg/mL post-dilution), respectively (Supplementary Table 2). As reported above, GML visibly precipitated at concentrations above 0.2 mg/mL in 0.9% saline. The increase in MEC of both LA and GML suggests that seminal plasma protects sperm against the immobilizing effects of these compounds; however, it is not sufficient to completely neutralize their activity.

#### 3.3.2 Time-Dependent Effect of LA and GML on Sperm Motility

**Figure 5.**
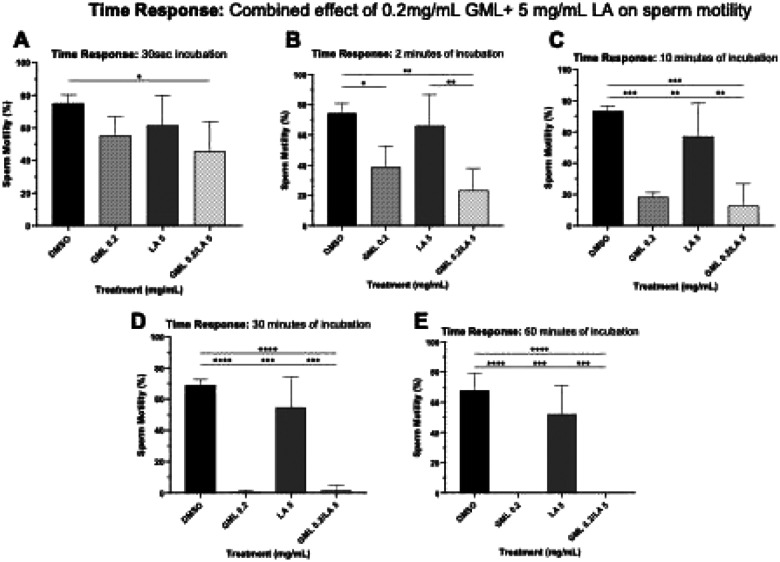
Time response assessment of combined sperm immobilizing effect of 0.2 mg/mL GML and 5 mg/mL LA. Sperm immobilizing effect of 0.2 mg/mL GML and 5 mg/mL LA at 30 secs, 2, 10, 30, and 60 minutes. Sperm motility was assessed by manual microscopic counting. Statistical analyses were conducted using one-way ANOVA with Tukey’s multiple comparison tests. All error bars represent SD (n=3). *p<0.05, **p<0.01, ***p<0.001, ****p<0.0001. DMSO 1:1000 (v/v) is the diluent control for 0.2 mg/mL GML.

**Figure 6.**
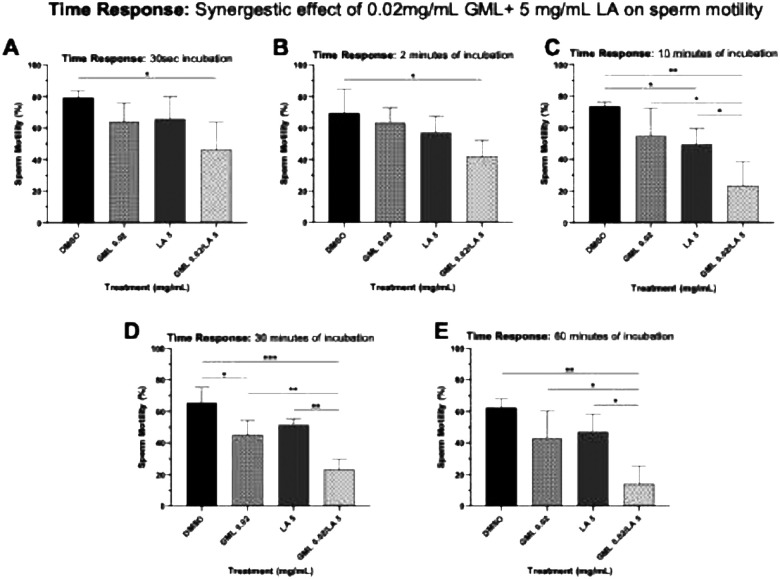
Synergistic effect of 0.02 mg/mL GML and 5 mg/mL LA on sperm motility. Combination treatment of 0.02mg/mL GML and 5mg/mL LA elicited a synergistic immobilizing effect on sperm at 30 secs, 2, 10, 30, and 60 mins following incubation. Sperm motility was assessed by manual microscopic counting. Statistical analyses were conducted using one-way ANOVA with Tukey’s multiple comparison tests. All error bars represent SD (n=3). *p<0.05, **p<0.01, ***p<0.001, ****p<0.0001. DMSO 1:10,000 (v/v) is the diluent control for 0.02 mg/mL GML.

A time-response study using increasing concentrations of LA and GML was conducted to determine the time needed to effect sperm-immobilization. Compounds were incubated with semen in a 1:1 dilution ratio considering the protective effects of seminal plasma. Similarly to the study above (Figs 3 and 4), LA and GML demonstrated a dose-dependent decrease in sperm motility (Figs. 5 and 6). The MEC of LA within 30 sec was 20 mg/mL (*p*= 0.0008), decreasing to 10 mg/mL (*p*= 0.0013) following 10 mins and persisting at that level during 120 mins of incubation (Fig. 5). At 20 mg/mL, GML took longer (10 min) to immobilize all sperm in the sample (10 min) (diluent control, *p*< 0.0001), but as incubation times increased (30–120 mins), it showed higher potency completely immobilizing sperm at 2 mg/mL (*p*< 0.0001, Fig. 6).

These results indicate that LA can induce immediate sperm immobilization at 10 mg/mL, while a lower steady concentration of GML at 2 mg/mL can maintain its inhibitory effect from 30–120 min following initial exposure.

#### 3.3.3 Synergistic Effect of LA/GML Combination Treatment

Combinational drug strategies often include compounds with different mechanisms of action that maximize efficacy, lower individual dosage, and minimize the risk of adverse effects. Combining NHC compounds can result in synergistic interactions that optimize their impact on sperm function. The combined effect of GML and LA (at suboptimal concentrations) on sperm motility was examined with a time-response assay.

**Figure 7.**
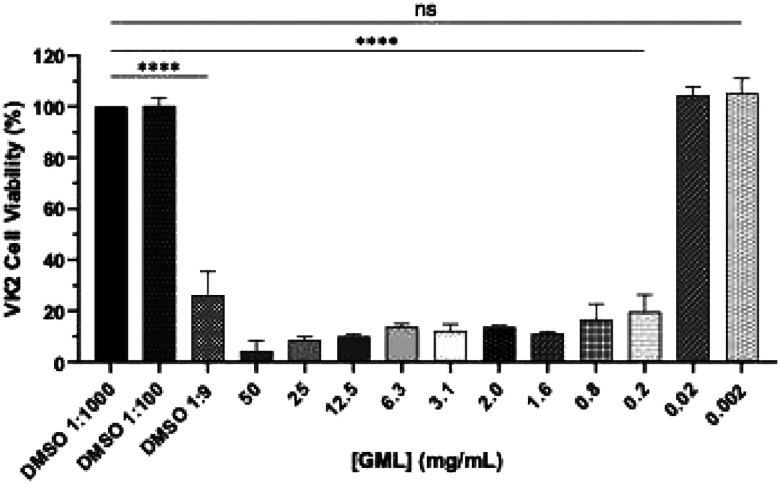
VK2 cell viability after 24hr GML exposure. Changes in cell viability of VK2 cells after 24 hrs exposure to varying concentrations of GML. % cell viability was determined via MTS assay. Statistical analyses were conducted using one-way ANOVA with Tukey’s multiple comparison tests. All error bars represent SD (n=3). **** *p*< 0.0001. DMSO 1:1000 (v/v), 1:100 (v/v), and 1:9 (v/v) served as diluent controls for 0.5, 5, and 50 mg/mL GML concentrations, respectively.

The initial combination of 0.2 mg/mL GML and 5 mg/mL LA demonstrated a clear increase in sperm immobilization effect within 20 mins of incubation compared to DMSO (diluent control, p=0.0029) and 5 mg/mL LA (p=0.0076). However, no difference was observed between the combined treatment of 0.2mg/mL GML and 5mg/mL LA and the single treatment with 0.2 mg/mL GML (p=0.2296). These results suggested that GML was driving the sperm immobilization activity in the combination treatment (Fig. 7).

**Figure 8.**
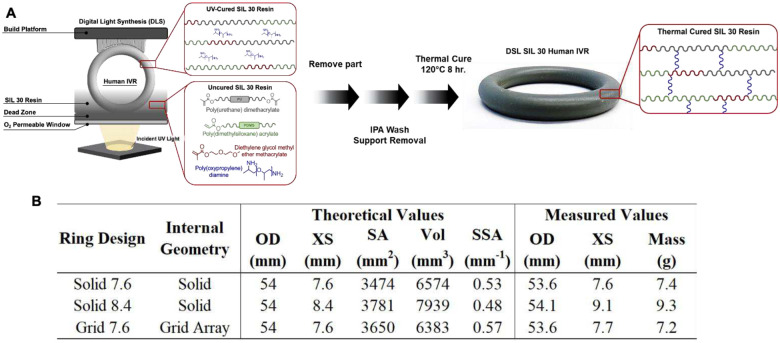
Overview of IVR fabrication. (A) Schematic of CLIP 3D-Printing. (B) Table summarizing ring design characteristics and metrics.

To further evaluate whether GML and LA synergistically inhibited sperm motility, sperm samples were treated with a lower suboptimal concentration of GML (0.02 mg/mL, Fig. 8). The combination treatment (0.02 mg/mL GML and 5 mg/mL LA) demonstrated a synergistic effect in inhibiting sperm motility within 10 min of incubation compared to DMSO (diluent control, *p*= 0.0012), 0.02 mg/mL GML (*p*= 0.0166), or 5 mg/mL LA (*p*= 0.0352), respectively. This synergistic effect was maintained throughout the 60 min incubation (*p*< 0.05). These findings point to the cooperative sperm immobilizing effects of GML and LA and suggest the benefits of formulating them as combinational drug strategies to elicit a more efficient contraceptive effect against human sperm.

#### 3.3.4 GML Induces Dose-Dependent Cytotoxicity in VK2 Cells

**Figure 9.**
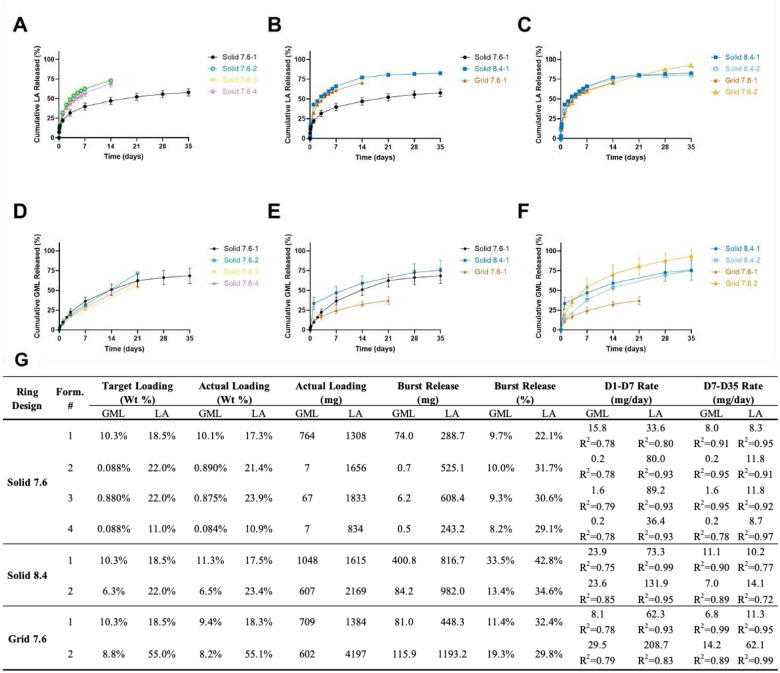
*In vitro* release studies of LA/GML IVRs. Comparing %LA released to assess effects on LA release kinetics through variations on (A) drug loading using Solid 7.6 rings, (B) ring design and metrics using equivalent LA wt% loading, and (C) increased surface area (relative to Solid 7.6, SA 3474 mm^2^). Comparisons of %GML released to assess effects on GML release kinetics through variations on (D) drug loading using Solid 7.6 rings, (E) ring design and metrics using equivalent LA wt% loading, and (F) increased surface area (relative to Solid 7.6, SA 3474 mm^2^). (G) Table summary of ring loading and *in vitro* release rates for all ring formulations.

Two-dimensional cell cultures using cell lines representative of respective organ systems are used to evaluate *in vitro* safety of compounds following prolonged (24-hour) exposures. To assess the impact of GML on the female genital tract (FGT), we used a human vaginal epithelial cell line (VK2/E6E7).^60^ VK2 cells were incubated with increasing concentrations of GML (0.002 to 50 mg/mL) for 24 hrs (Fig. 9). GML at concentrations ≥ 0.2 mg/mL significantly decreased cell viability, compared to vehicle control (DMSO 1:100, *p*< 0.0001). Concentrations of GML ≤ 0.02 mg/mL (*p*= 0.805) were considered non-cytotoxic in VK2 cells following prolonged incubation. LA could not be tested in this assay due to its pH-lowering effects.

Given the combined efficacy and cytotoxicity results from the cell-based assays, additional IVR formulations (Solid 7.6–2, 3, and 4) were developed to target *in vitro* release rates of 38–75 mg/day for LA and 0.15–1.5 mg/day for GML. While these findings confirm the contraceptive effects of these anti-sperm compounds, assessing the safety of both LA and GML in tissue explants representative of the FGT and animal models is paramount. Future studies will also include in vivo safety and efficacy studies in the ovine model to determine the optimal LA/GML doses that are safe and effective in post-coital sperm motility studies.

### 3.4 Fabrication of 3D-printed IVRs with Continuous Liquid Interface Production (CLIP)

**Figure 10.**
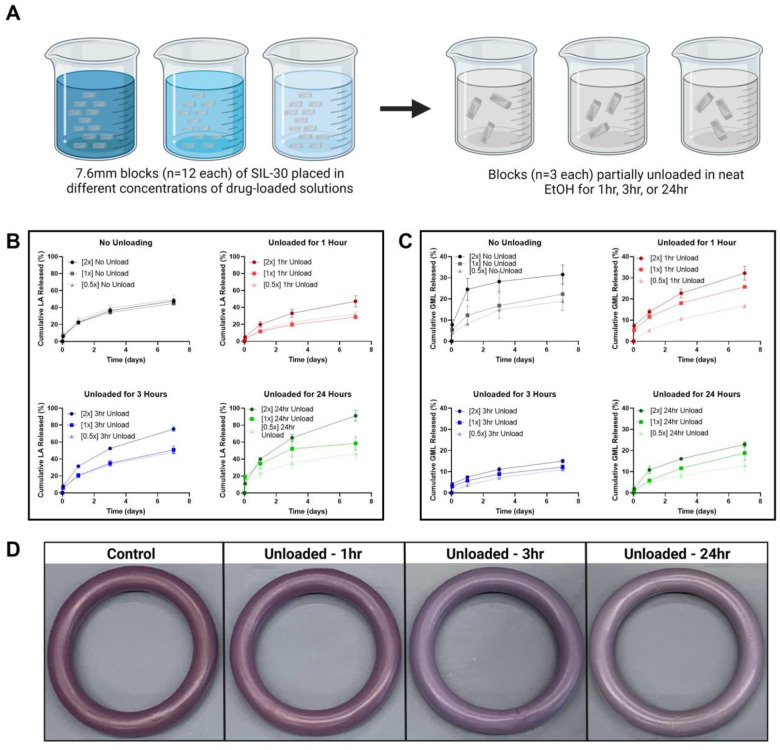
Partial unloading of LA and GML. (A) Schematic of partial unloading procedure. (B) Precent (%) LA released from unloaded blocks. (C) Percent (%) GML released from unloaded blocks. (D) Visual representation of partial unloading at different timepoints using IVRs loaded with rhodamine B as a model hydrophobic dye.

Three different intravaginal ring designs were developed and fabricated in SIL 30 using CLIP 3D-printing (Fig. 10A). Using computationally aided design (CAD) allowed for the rapid manipulation of IVR mass, surface area, volume, and drug diffusion distances which impact drug loading and release kinetics. Two of the rings were monolithic solids of varying dimensions (Solid 7.6, Solid 8.4) and the third ring was designed to mimic the dimensions of the solid 7.6 IVR while incorporating geometric complexity (Grid 7.6). The outer diameters of all ring types were set at 54 mm, equivalent to the marketed NuvaRing^®^ hormonal IVR.^7–9^ The specific dimensions and characteristics of each ring type are illustrated in Fig. 10B.

### 3.5 Development of Weight-Based Linear Loading Equations

**Figure 11.**
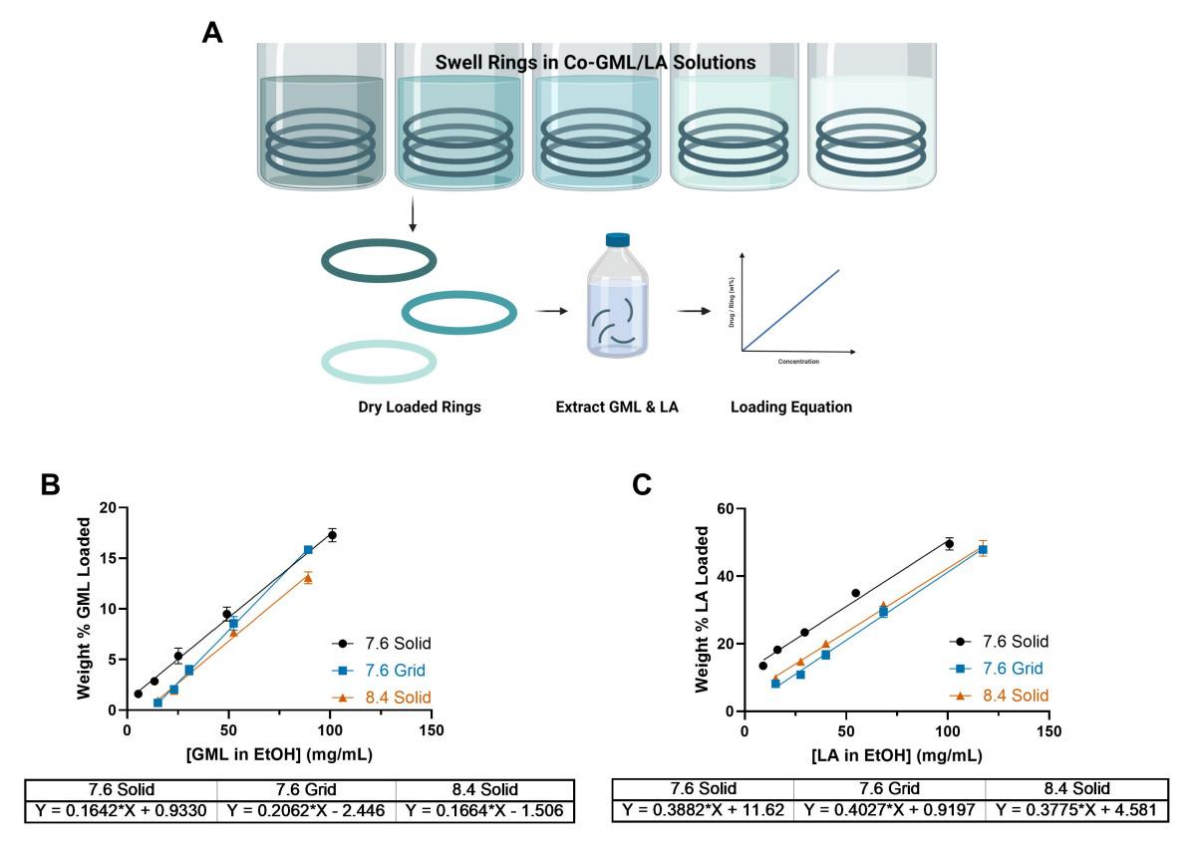
Post-fabrication drug loading and loading equations. (A) Schematic of post-fabrication drug loading process in serially diluted drug concentrations for the development of weight-based loading equations. (B) GML weight-based loading equations. (C) LA weight-based loading equations.

GML and LA were co-loaded in the finished IVRs through absorption by swelling the rings in an EtOH solution containing both compounds (Fig. 11A). The silicone polyurethane of SIL-30 possesses a hydrophobic backbone which enables maximal swelling in organic solvents. Loading solutions were prepared in EtOH due to its high swelling capacity (316–365% swelling) and its designation as a Class 3 residual solvent, indicating its low safety risk.^53^

Serially-diluted loading solutions were prepared to develop linear weight-based loading equations for each ring type utilized (Fig. 11B&C).^21,25,48^ These loading equations were utilized to incorporate target amounts of drug homogenously into the rings. Notably, IVRs loaded in solutions of lactic acid above 300 mg/mL elicited an undesirable tacky feel which set an upper limit for LA loading (300 mg/mL LA in EtOH loading solution) in future experiments.

### 3.6 In vitro Drug Release Studies

A total of eight (8) ring formulations were created from the three ring designs (Solid 7.6, Solid 8.4, Grid Array 7.6) and varied drug loading as described in **Fig. 12G**. This library of designs allowed for both ring characteristics (e.g. surface area, volume, and geometric complexity) and drug loading to be investigated for their effects on drug release kinetics. *In vitro* release studies were carried out in SVF (25 mM sodium acetate, 2% solutol, pH 4.2) to assess the release of LA and GML over ≥30 days. This time frame provides a clinically relevant replacement frequency of existing intravaginal contraceptive rings containing combination hormones, such as the NuvaRing^®^.^7,8^ Initial formulations (Solid 7.6–1; Solid 8.4–1,−2; and Grid 7.6–1,−2) were designed to target release rates of 2.5–25 mg GML/day and 14–45 mg LA/day while later designs (Solid 7.6–2,−3,−4) targeted amended release rates of 0.15–2.5 mg GML/day and 38–75 mg LA/day. To investigate the effect of ring design on drug release kinetics, direct comparisons between ring types were made by loading each with 10.3 wt% GML and 18.5 wt% LA. A second loading profile was generated for both the Solid 8.4 and Grid 7.6 ring types to assess the effect of drug loading on release rates. Lastly, three additional ring formulations were created using the Solid 7.6 ring design with drug loading and release targets that were informed by cell-based efficacy and toxicity assay results (Section 4.2.3). These *in vitro* release studies highlight the design and drug loading flexibility of CLIP 3D-printed IVRs and the ability to co-load and co-deliver two compounds with highly different lipophilicities.^49^

When comparing the Solid 7.6 rings **(Fig. 12A&D)**, all formulations displayed comparable burst released for GML (8.2–10%) within the first 24 hours. A similar pattern was observed in the burst release of LA between Solid 7.6–2 (31.7%), Solid 7.6–3 (31.7%), and Solid 7.6–4 (29.1%). A lower LA burst was observed with Solid 7.6–1 (22.1%).

Furthermore, increasing IVR drug loading generally resulted in a corresponding increase in drug release rates. This was demonstrated across the Solid 7.6 rings, with initial GML release rates (d1-d7) increasing from 0.2 mg/day (0.088 wt% GML, Solid 7.6–2,4) to 1.6 mg/day (0.88 wt% GML, Solid 7.6–3) and 15.8 mg/day (10.3 wt% GML, Solid 7.6–1). Similarly, within the first week (d1-d7), LA release rates increased from 36.4 mg/day for Solid 7.6–4 IVR (11 wt% LA) to 80.0 and 89.2 mg/day for Solid 7.6–2 and Solid 7.6–3 IVRs (22 wt% LA). Interestingly, this pattern was not sustained by the Solid 7.6–1 IVR which had a LA loading of 18.5 wt% and only released 33.6 mg/day in the first week (d1-d7). In other controlled release formulations, the release of hydrophilic compounds has been observed to slow down upon inclusion of a hydrophobic compound.^61–63^ As Solid 7.6–1 was formulated with a high concentration of GML (764 mg), a hydrophobic compound,^64^ the divergences in LA burst and release rates described above may be due to this interactive effect. Further studies should be conducted to determine the strength of this effect in the SIL-30 matrix.

In rings with equivalent wt% of loaded drug **(Fig. 12B&E)**, a small increase in surface area created by geometric complexity (Grid 7.6, SA = 3650 mm^2^) or thicker cross-sectional diameters (Solid 8.4, SA = 3781 mm^2^), resulted in increased burst release of both compounds when compared to Solid 7.6 (SA = 3474 mm^2^). The effect of surface area became more apparent after the initial burst release, with higher LA release rates observed with increasing ring surface area (33.6 mg/day Solid 7.6–1, 62.3 mg/day Grid 7.6–1, 73.3 mg/day Solid 8.4–1) in the first week. Greater surface area increases the contact between drug-loaded IVRs and SVF. As such, greater drug release rates can occur and is likely to be more prominent for hydrophilic compounds.

Lastly, the geometric complexity introduced in the RA Grid ring appeared to reduce the plateauing effect observed with LA in the first week and elicited more sustained release over 35 days **(Fig. 12C&F)**. The solid cross-section present in existing, FDA-approved IVRs prevents complete drug diffusion resulting in incomplete drug release (<50%) during the treatment duration.^65,66^ As a result, excess drug is loaded in solid rings to ensure release is sustained at therapeutic levels. A similar plateau was observed in the Solid 7.6 and Solid 8.4 rings with LA release not reaching completion (100% release). In contrast, the release of LA from Grid 7.6 IVRs elicited a sustained cumulative release profile and near complete drug release, demonstrating the ability to enhance drug release kinetics and reduce drug waste via geometric complexity enabled by 3D printing.

Altogether, these *in vitro* release results have further demonstrated the mechanisms of drug release from CLIP 3D-printed IVRs and the ability to fine-tune drug release kinetics by varying drug loading, surface area, and/or introducing geometric complexity.

### 3.7 In vitro release post burst release via partial drug unloading

Drug release from silicone-based matrix IVRs is known to mainly happen through diffusion of the API from the matrix.^67^ As a result, drug gradients closest to the surface of an IVR can be released rapidly during the initial phase of release. This effect is commonly known as burst release and is illustrated by the first 24-hours of incubation in this study.^68^ A burst release is commonly seen with IVRs and other delivery systems, and can be relatively high for hydrophilic compounds like lactic acid.^68,69^ As a result, a burst may cause elevated drug exposure and possible safety concerns. A high initial burst can also result in reduced release durations and potentially reduced efficacy.

In consideration of this, many researchers have sought to reduce or eliminate burst release. One previously employed method reported by Lee *et al*.^70^ subverted the traditional preference for homogenously distributed drug by extracting surface drug prior to release from a hydrogel. The resultant ‘non-uniform drug distribution’ created an artificial, and temporary, increase in diffusion distance which could reduce the overall burst release.

A similar extraction method was explored here with GML and LA in the SIL 30 3D-printed blocks, referred to as “partial unloading” **(Fig. 13A)**. Scaled rectangular blocks of SIL 30 were 3D-printed and cured to act as a simplified and efficient model for the IVRs.^25^ An initial loading solution of GML and LA was prepared targeting 2× the concentration utilized to load Solid 7.6–1. Two serial dilutions were performed to create a total of three loading solutions (designated as 2×, 1×, and 0.5×) to examine the effects of partial unloading over a range of concentrations. Twelve (12) blocks were placed in each loading solution and allowed to swell for 48 hours to reach equilibrium swelling. Drug concentrations within the blocks were linearly correlated to drug concentration in the loading solutions.^25^ After loading and subsequent air-drying, each set of blocks (n=12) was separated into four groups (n=3): No unloading (0-hour), 1-hour unloading, 3-hour unloading, and 24-hour unloading. The blocks were placed in neat EtOH for their designated time-period to extract surface drug. Following drug unloading and air-drying, blocks were individually incubated in SVF to assess the effect of partial unloading on in vitro release.

As expected, control blocks that were incubated in more concentrated loading solution elicited higher burst (Supplementary Figure 3) and higher overall drug release rates. When examined relative to their total loading, the percent drug released during the initial 24-hour burst phase decreased as the loading concentration decreased (**Fig. 13**). After the 24-hour burst, the daily percent drug released from the control (no unloading) blocks were similar despite their disparities in initial drug loading.

For blocks loaded in the same solution, the amount of drug released during the initial 24-hour burst phase decreased as unloading time increased. For example, blocks loaded in the 2× solution displayed an average burst release of 42.4 mg LA and 87.2 mg GML without any unloading time. With unloading times of 1hr, 3hr, and 24hr, the burst release decreased to 23.6, 19.8, and 16.4 mg LA and 39.6, 19.2, 18.0 mg GML respectively (Supplementary Fig. 3). On the other hand, the percent burst release of LA and GML did not follow a similar correlation to the loading solution concentration. For example, blocks loaded in the 2× solution elicited an average burst release of 24.5% GML and 22.4% LA. After 1hr of unloading, the burst release decreased to 13.9% GML and 19.8% LA. On the other hand, blocks loaded in the 2× solution and were unloaded for 3 hours elicited an even lower burst release for GML (7.9%), but a higher burst release for LA (31.4%). Blocks loaded in the 2× solution and were subsequently unloaded for 24 hours elicited a higher burst released for LA (40.0%) and for GML burst (10.2%). These results can be attributed to the fact that short extraction durations allow surface drug to diffuse without allowing enough time for redistribution of remaining drug. The impact of the length of time is likely exacerbated by the physiochemical properties of the dispersed compounds. As the percent of burst release of LA was lowest (13.0– 19.8%) with only 1hr of unloading and the percent burst release of GML was lowest (3.7 – 8.5%) after 3hrs of unloading, it is likely the hydrophilic LA achieves an equilibrium in solution more rapidly than the hydrophobic GML.

After the initial 24 hour burst phase, GML release rates between days 1 and 7 were not significantly different in most blocks (averaging 1.6% GML per day, [p=0.65, one-way ANOVA, GraphPad Prism], Supplementary Fig. 3). and 4.5% LA per day, Within this same time period, LA release rates averaged 4.5% LA per day, though blocks with the highest initial LA loading (2×) elicited a significantly increased percent drug release as unloading time increased (8.2% per day, 24hr unload, [p=0.018, one-way ANOVA, GraphPad Prism]).

The effects of partial unloading were visually illustrated using two model fluorescent dyes, rhodamine B (hydrophobic) and Nile blue A (hydrophilic), as shown in **Fig. 13D**. The fluorescent intensity of each dye was measured in samples of the ethanol solutions used for partial unloading (Supplementary Figure 4). Higher concentrations of Nile blue A were released during the unloading step compared to rhodamine B due to its hydrophilic nature.

Collectively, these results illustrate ‘partial unloading’ as a potential tool for the reduction of burst release from our 3D-printed IVRs. Future research will investigate the effects of the unloading method on burst reduction using human-sized rings to establish its feasibility as a functional framework for future use in product development.

## 4. CONCLUSION

In summary, our results demonstrated the successful co-formulation and delivery of two nonhormonal contraceptive compounds, LA and GML, at or above target release rates for ≥30 days *in vitro*. Through different mechanisms of action, LA and GML were able to quickly and persistently immobilize human sperm in semen in a dose- and time-dependent manner. The release rates needed to achieve this activity were attained by adjusting the engineering process of the 3D-printed IVR. These results support the potential for a user-controlled nonhormonal reversible contraceptive with a monthly duration of action, addressing a major gap in existing contraceptives and subsequently helping to reduce the rates of unintended pregnancies. Future studies will investigate *in vivo* safety and efficacy in relevant models and the effect of GML/LA as both a contraceptive and an HIV pre-exposure prophylactic (PrEP) combination to explore the potential of a multi-purpose prevention technology (MPT) IVR.

Furthermore, this work has provided greater insights into the fundamental mechanisms of drug release from CLIP 3D-printed IVRs. Increasing ring surface area resulted in greater drug release, the effects of which were intensified for the hydrophilic compound, LA. It was further demonstrated that geometric complexity, a feature ascribed to fabrication by 3D-printing, resulted in enhance drug release rates and near complete release of LA and GML. Moreover, results above from the *in vitro* release studies showed that interactions between the co-loaded compounds may impact drug release. Future studies should be conducted to fully characterize the effects of drug physiochemical properties in the SIL 30 matrix. Overall, these results support the rational design of future single and co-loaded IVRs for nonhormonal contraception.

## Supplementary Material

1

## Figures and Tables

**Figure 1.**
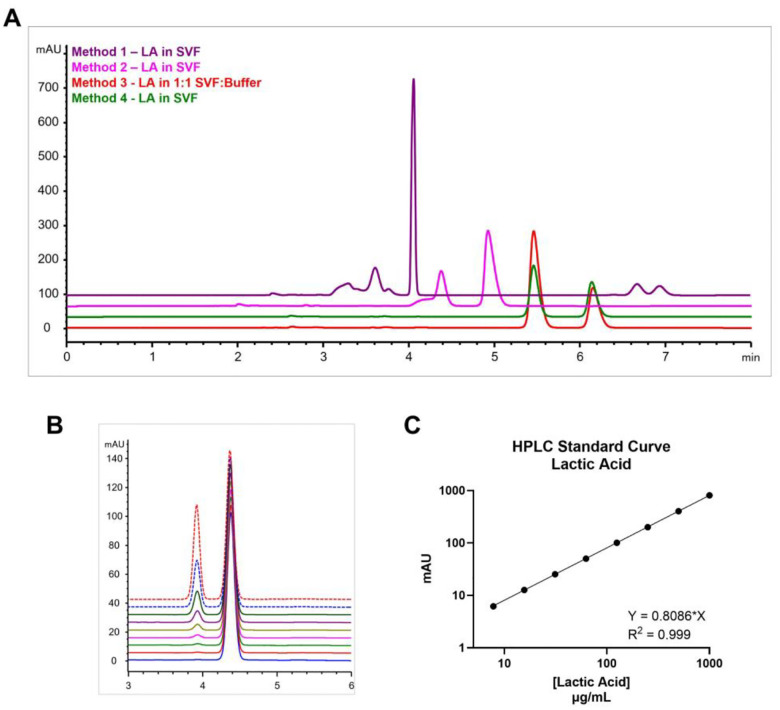
HPLC method development for LA. **(A)** Results of HPLC method development to quantify LA via separation of LA and acetic acid, a component of SVF. **(B)** HPLC traces of LA standards, prepped in 1:1 SVF:10 mM phosphate buffer. All traces offset by 5%. **(C)** Standard curve generated using optimized separation method for HPLC quantification of LA.

**Figure 2.**
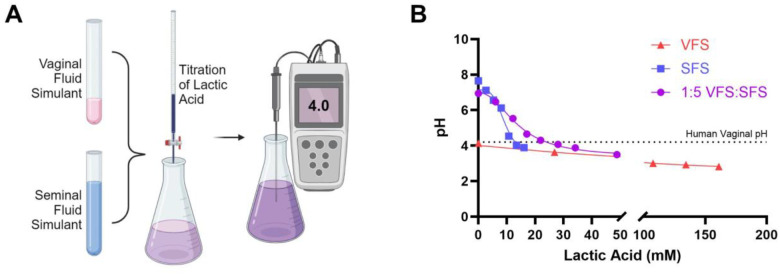
Titration of LA to establish target loading. **(A)** Schematic representing titrations performed to establish an initial target daily dose of LA for vaginal acidification. **(B)** Results of LA titration in SVF alone, SFS alone, and in a 1:5 VFS:SFS solution.

**Figure 1.**
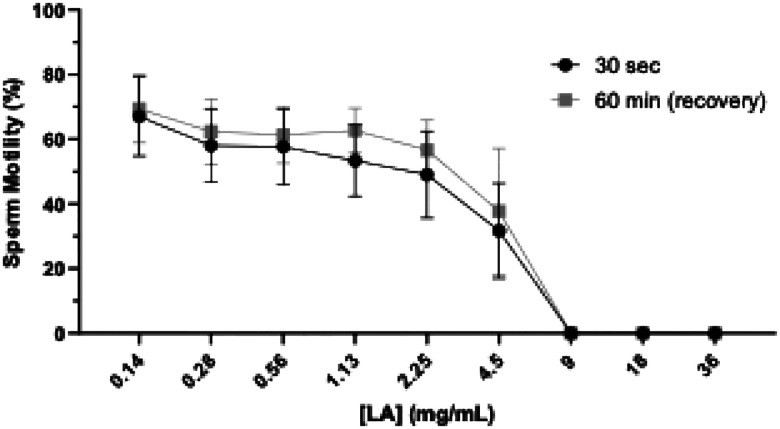
Sperm immobilizing activity of LA and recovery of sperm motility. Sperm immobilization activity of LA within 30 sec of incubation and recovery of sperm motility following 60 mins with Ham’s F-10/1% HSA media and 60 min additional incubation (sperm recovery period). Sperm motility was assessed by manual microscopic counting. Statistical analyses were conducted using two-way ANOVA with Tukey’s multiple comparison tests. All error bars represent SEM (n=3).

**Figure 2.**
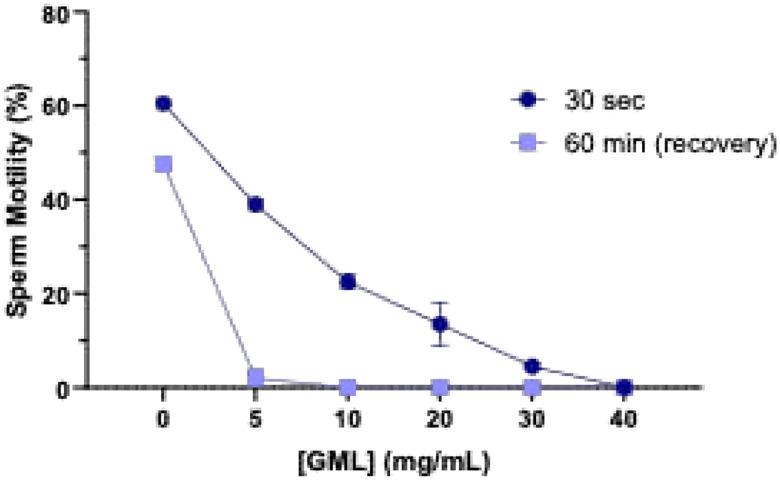
Sperm immobilizing activity of GML & recovery of sperm motility. Sperm immobilization activity of GML within 30 sec of incubation and following dilution with Ham’s F-10/1% HSA media and an additional 60 min incubation (sperm recovery period). Sperm motility was assessed by manual microscopic counting. Statistical analyses were conducted using two-way ANOVA with Tukey’s multiple comparison tests. All error bars represent SEM (n=3). 0 mg/mL represents DMSO 1:8 (v/v) as the diluent control for 40 mg/mL GML.

**Table 1.** Saturation solubility of GML in relevant solvents

Solvent	GML Saturation Solubility (mg/mL)
Acetone	255.2 ± 1.74
MeOH	277.86 ± 45.17
SVF (w/ 2% solutol)	3.25 ± 0.45
EtOH	271.15 ± 18.02
Water	4.48 ± 0.12

**Table 2.** Assessing sperm immobilizing activity and reversibility of LA and GL using Sander-Cramer assay.

	1:6 (Semen):(Compound)	
	MEC ± SD (mg/mL)*	MEC after recovery ± SD (mg/mL)^†^
**D/L-Lactic Acid**	9	9
**GML**	45 ± 5.5	1.7 ± 0.9^γ^
**N-9**	0.125	0.125

* Minimum effective concentration (MEC) determined after initial 30 sec exposure to compounds in 1:6 dilution ratio (50 μL semen + 250 μL compound)

† Recovery of sperm immobilization activity was determined by diluting the treated sample for 60 mins with 1 mL of inhibitor-free media

γ Visible Precipitate

## Data Availability

The datasets generated during and/or analyzed during the current study are available from the corresponding author on reasonable request.

## Data availability statement:

Data will be made available from the corresponding author upon reasonable request.
