# Supplementary material for: First-in-line 3D-printed intravaginal ring for nonhormonal contraception
Source: Res Sq. 2025 Nov 21:rs.3.rs-7935107. Preprint. [Version 1] doi: 10.21203/rs.3.rs-7935107/v1 (PMC12668138; doi:10.21203/rs.3.rs-7935107/v1)
Supplement: 1 [file NIHPPRS7935107V1-supplement-1.pdf]

SUPPLEMENTAL INFORMATION

Supplemental Table 1. Gradient method developed for LC-MS quantification of GML in solution.

Time (min)	Water (w/0.1% formic acid)	ACN (w/0.1% formic acid)
0	95	5
1	95	5
3	0	100
5	0	100
6	95	5
8	95	5

Supplemental Table 2. Assessing sperm immobilizing activity and reversibility of LA and GL using modified Sander-Cramer assay.

	1:1 (Semen):(Compound)	
	MEC ± SD (mg/mL)*	MEC after recovery ± SD (mg/mL)†
**D/L-Lactic Acid**	18	15 ± 5.2
**GML**	55 ± 5.8$^{\gamma}$	1.7 ± 0.9$^{\gamma}$

*Minimum effective concentration (MEC) determined after initial 30 sec exposure to compounds in 1:1 dilution ratio (50 µL semen + 50 µL compound)

†Recovery of sperm immobilization activity was determined by diluting treated sample for 60 mins with 1 mL of inhibitor-free media

$^{\gamma}$Visible Precipitate

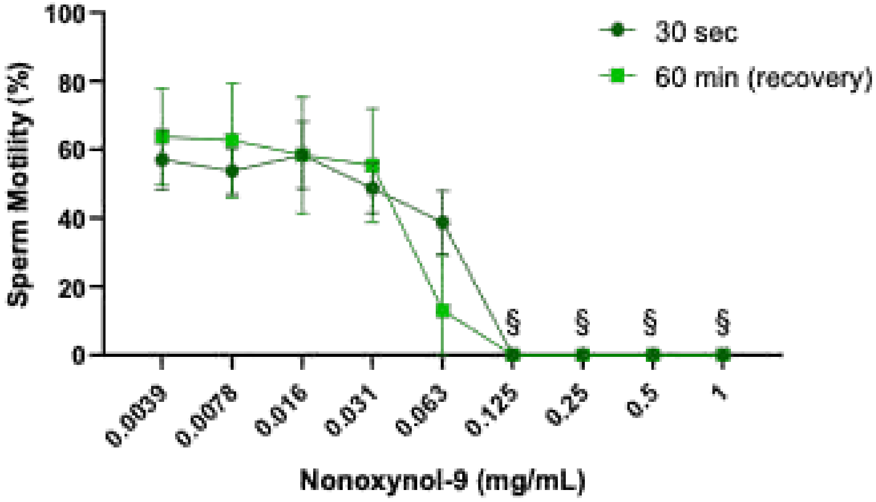

Supplemental Figure 1. Sperm immobilizing activity of N-9 and recovery of sperm motility. Sperm immobilization activity of N-9 within 30 sec of incubation and following dilution with Ham's F-10/1% HSA media and an additional 60 min incubation (sperm recovery period). Sperm motility was assessed by manual microscopic counting. Statistical analyses were conducted using two-way ANOVA with Tukey's multiple comparison tests. All error bars represent SEM (n=3). § *p*<0.0001, represents comparison of sperm motility between concentrations of 0.125-1 mg/mL with 0.039-0.063 mg/mL following 30 secs and 60 min (recovery post-dilution).

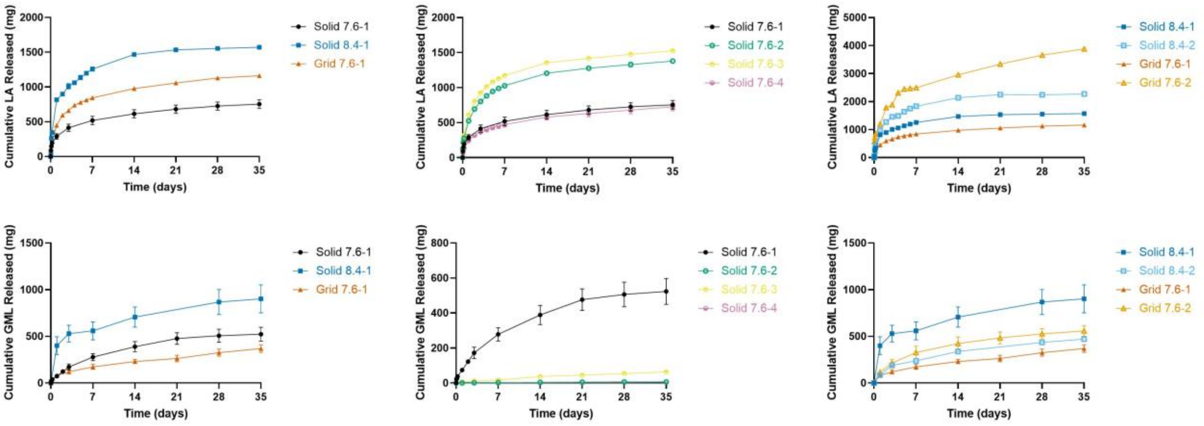

Supplemental Figure 2. Cumulative in vitro drug release (mg) from IVRs. In vitro release studies of non-hormonal IVR formulations comparing mg LA released to display effects on LA release kinetics through (a) drug loading using Solid 7.6 rings, (b) ring design and metrics using equivalent LA wt%, (c) increased surface area (relative to Solid 7.6, SA 3474mm^2). Additionally, figure includes comparisons of mg GML released to display effects on GML release kinetics through (d) drug loading using Solid 7.6 rings, (e) ring design and metrics using equivalent GML wt%, (f) increased surface area (relative to Solid 7.6, SA 3474 mm^2). Table (g) summary of ring loading and in vitro release rates for all ring formulations.

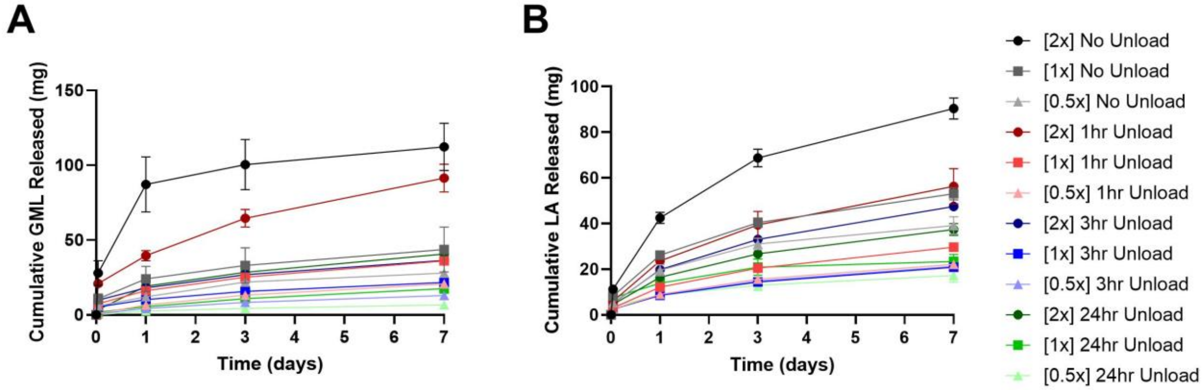

C

Load Sol'n	Unload Time (hrs)	Amount Loading (mg)		Burst Release (mg)		Burst Release (%)		D1-D7 Rate (mg/day)		D1-D7 Rate (%/day)	
		GML	LA	GML	LA	GML	LA	GML	LA	GML	LA
[2×]	0	356	189	87.2	42.4	24.5%	22.4%	4.0 R^2=0.95	7.6 R^2=0.94	1.1 R^2=0.95	4.0 R^2=0.94
	1	284	119	39.6	23.6	13.9%	19.8%	4.8 R^2=0.97	5.3 R^2=0.97	1.9 R^2=0.97	4.4 R^2=0.97
	3	242	63	19.2	19.8	7.9%	31.4%	3.0 R^2=0.97	4..5 R^2=0.97	1.2 R^2=0.97	7.1 R^2=0.97
	24	177	41	18.0	16.4	10.2%	40.0%	3.5 R^2=0.99	3.4 R^2=0.97	2.0 R^2=0.99	8.2 R^2=0.97
[1×]	0	196	118	24.1	26.1	12.3%	22.1%	3.2 R^2=0.98	4.3 R^2=0.95	1.6 R^2=0.98	3.6 R^2=0.95
	1	140	104	16.2	13.9	11.6%	13.4%	3.2 R^2=0.98	2.8 R^2=0.97	2.3 R^2=0.98	2.7 R^2=0.97
	3	120	57	10.2	12.1	8.5%	21.2%	1.8 R^2=0.97	2.0 R^2=0.97	1.0 R^2=0.97	4.9 R^2=0.97
	24	93	40	5.4	8.5	5.8%	21.3%	2.0 R^2=0.98	1.5 R^2=0.80	2.1 R^2=0.98	3.6 R^2=0.80
[0.5×]	0	147	79	12.1	19.6	8.2%	24.8%	2.5 R^2=0.89	3.1 R^2=0.92	1.7 R^2=0.89	3.9 R^2=0.92
	1	124	69	6.5	9.0	5.2%	13.0%	2.3 R^2=0.98	2.1 R^2=0.96	1.9 R^2=0.98	3.1 R^2=0.96
	3	117	44	4.3	8.7	3.7%	19.8%	1.4 R^2=0.98	2.0 R^2=0.97	1.2 R^2=0.98	4.6 R^2=0.97
	24	52	37	2.5	8.7	4.8%	23.5%	0.7 R^2=0.99	1.4 R^2=0.96	1.3 R^2=0.99	3.7 R^2=0.96

Supplemental Figure 3. In vitro release from unloaded blocks. Details of (a) cumulative in vitro release (mg) from unloaded blocks and (b) summary table of block loading and release rates.

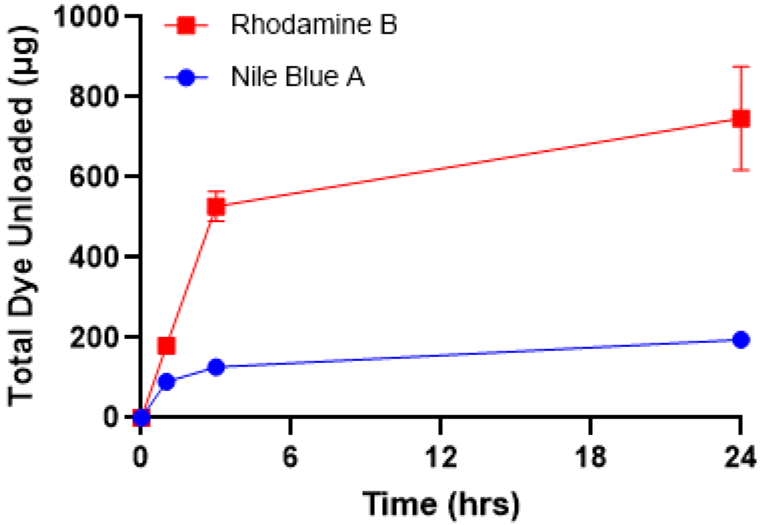

Supplemental Figure 4. Partial unloading of fluorescent dyes. Quantification of dye removed from IVRs due to time-dependent partial unloading in EtOH.
